# Aquality32: A low-cost, open-source air quality monitoring device leveraging the ESP32 and google platform

**DOI:** 10.1016/j.ohx.2024.e00607

**Published:** 2024-11-12

**Authors:** Daniel M. Pineda-Tobón, Albeiro Espinosa-Bedoya, Jhon W. Branch-Bedoya

**Affiliations:** Facultad de Minas, Universidad Nacional de Colombia – Sede Medellín, Colombia

**Keywords:** IoT, ESP32, Air quality, PM2.5, CO_2_

## Abstract

We present AQuality32, an open-source air quality monitoring device designed to address the challenges faced by small research teams in accessing affordable and versatile air quality monitoring solutions. The device utilizes an ESP32 System on Module (SoM) and integrates sensors for CO_2_ (SDC30 from Sensirion) and particulate matter (HM3301 from Seeed) measurement, along with temperature, relative humidity, and extensive sensing possibilities. It can be powered by a battery and is suitable for both stationary and on-the-go measurements. The paper details the hardware description, building instructions, programming, calibration procedures, and data collection setup for AQuality32. Validation experiments assess communication stability, geolocation accuracy, and environmental monitoring capabilities. The results demonstrate the device’s reliability, affordability, and suitability for various applications in environmental sciences, public health, and indoor/outdoor air quality monitoring. The paper emphasizes the importance of robust solutions, openness, and easy documentation for widespread adoption and impact in air quality research and monitoring.

## Graphical abstract

**Figure:**
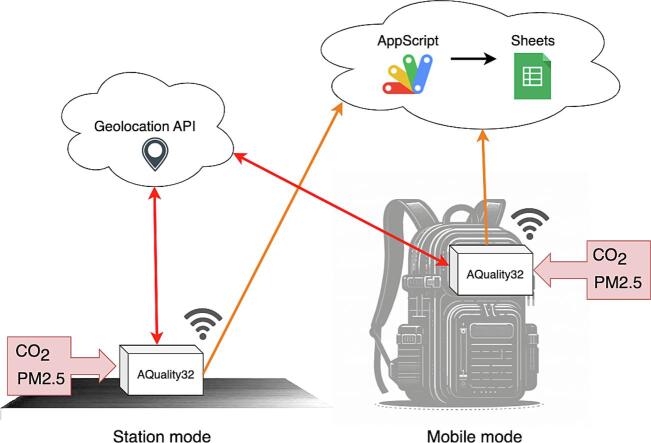

Specifications table

**Table:** 

Hardware name	AQuality32
Subject area	• Environmental, planetary and agricultural sciences • Educational tools and open source alternatives to existing infrastructure • General
Hardware type	• Field measurements and sensors • Electrical engineering and computer science
Closest commercial analog	No commercial analog is available.
Open source license	GNU General Public License (GPL)
Cost of hardware	170USD
Source file repository	https://doi.org/10.17605/OSF.IO/N9R5B

## 1 Hardware in context

Air quality monitoring and its implications have garnered increased attention in recent years due to concerns regarding public health and on indoor environments poorly ventilated it can be especially harmful for vulnerable groups such as elderly, children and in general people with respiratory health issues [1]. However, conducting air quality research often necessitates access to equipment that can be challenging to obtain or even tedious and time-consuming chemical analyses, particularly for small teams and researchers in resource-constrained regions. Some authors highlight the usage of low-cost sensors, which comes with limitations compared to expensive devices but have added benefits, mainly related to the possibility of higher spatial resolution and mobility [2].

While several air quality devices are available on the market, they frequently come with a hefty price tag and limited functionalities [3]. Even commercial “affordable” devices are relatively expensive, for instance, the pSense AZ-0002 portable CO_2_ meter is considered one of the more affordable options, priced at approximately 380 USD. In contrast, the aeroqual S-500, another portable device priced at over 1000 USD, can measure CO_2_, particulate matter, and additional parameters but requires separate accessories for different measurements, substantially increasing its overall cost.

Alternatively, there are sensors that can be combined with open-source technologies such as Arduino, Raspberry and even bare microcontrollers significantly lowering the price tag. Unfortunately, the implementation of these sensors often requires advanced knowledge in electronics, programming, and design, which can significantly difficult their usage for most researchers. For instance, Khan et al. [4] developed a cost effective implementation similar to the one developed on this project, however, their solution intends to measure other variables, lacks printed circuit board (PCB) design and a case to ensure robustness and relies on an ESP32 development board with no battery. On the other hand, Chaoraingern et al. [5] present an interesting implementation that includes an ultra-low-cost CO_2_ sensor and a particulate matter sensor cleverly installed on top of a vacuum cleaner for indoor air quality monitoring, however, their implementation lacks details for accurate reproduction or missing information, which applies for both cited authors. The mentioned systems are not openly presented which difficult to reproduce their solutions.

On the open-source side, a notable development is the SentinAir platform, a modular system based on the Raspberry Pi single board computer that supports a wide range of sensors and can be used for both portable air quality monitoring and on-field calibration of low-cost gas sensors [6], [7]. However, this development is still in an early prototype stage and lacks a printed circuit board to ease its construction. It also requires a certain level of skill to operate, as it necessitates having the Raspberry Pi properly configured and the custom software set up.

For intensive usage of these devices on field and laboratory it is highly recommended to implement a robust solution while at the same time encourage openness and easy to read documentation. This way, anyone can better benefit from these developments. The device proposed in this work, from now on called AQuality32 is like SentinAir in that it is based on a low-cost, open-source platform and supports a variety of sensors. However, AQuality32 is different in that it uses the ESP32 microcontroller, which is more power-efficient and cost-efficient than the Raspberry Pi and is considerably easier to reproduce since a custom circuit printed board is provided. Additionally, our work focuses on the use of Google services for data collection and geolocation. AQuality32 is capable of measuring CO_2_, particulate matter (PM2.5, PM1, and PM10), relative humidity, and temperature, but the user can also add additional sensors if required. It also features Wi-Fi and Bluetooth connectivity for easy data transmission. Additionally, the device can be powered by a battery, making it portable and suitable for on-the-go and stationary measurements. Just in terms of materials, this device can cost around 170USD, making it ideal for researchers with a low budget and scalable for those who wish to cover large spaces.

## 2 Hardware description

**Fig. 1:**
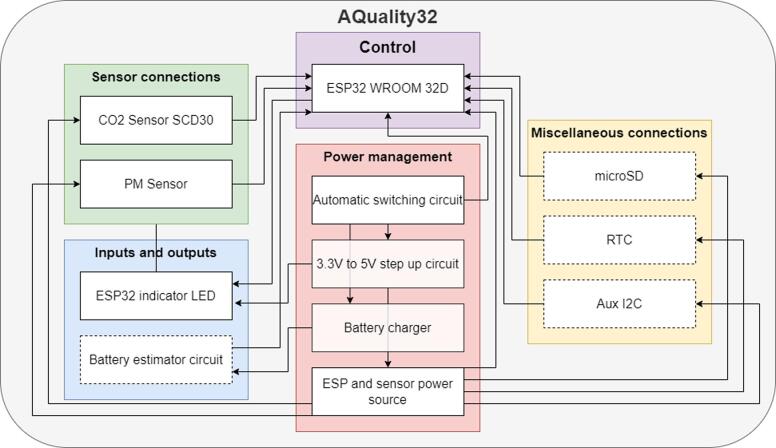
AQuality32 hardware block representation.

The proposed air quality monitoring device utilizes an ESP32 System on Module (SoM), offering a robust platform for embedded systems development and connectivity. This feature is advantageous for developers seeking hardware and firmware customization. The ESP32 SoMs support programming through the official Espressif IoT Development Framework (IDF), as well as compatibility with the Arduino framework and Micropython, providing accessible programming environments for makers. In this project, the firmware was developed using Platform IO on Visual Studio Code, employing the Arduino framework and FreeRTOS. This approach serves as a customizable example tailored to specific project requirements. While the device supports additional functionalities such as microSD module integration, real-time clock (RTC), and GPS connectivity by using the auxiliary I^2^C port, the focus here is on cost-effectiveness, utilizing essential components only. Fig. 1 represents the essential hardware blocks which can also be found with the same names on the KiCAD provided designs. The arrows are a simplified input/output relationship between blocks only to indicate which blocks are linked, for instance, the “ESP32 indicator LED” is represented as an output for the “ESP32 WROOM 32D”.

The dashed lined blocks were not implemented in this work. The following table includes a simple description of the blocks on Fig. 1: (See Table 1)

**Table 1:** Hardware block description.

**Section**	**Block**	**Description**
Control	ESP32 WROOM 32D	Includes a simple circuit to connect and program the ESP32, additionally adds some pull up resistors for the I2C interface.
Power management	Automatic switching circuit	Allows to power de device directly from a power adapter and cut the power coming from the battery. Additionally, since the microUSB is used for both programming and power/charging, it includes the connection to RX and TX for the ESP32 programming.
	3.3 V to 5 V step up circuit	Allows to take the theoretical 3.3 V from the battery and convert it to 5 V
	Battery charger	A circuit to charge the battery and indicate when it is fully charged.
	ESP and sensor power source	To power the circuit to a stable 3.3 V, an LDO is used to convert the 5 V either from the battery or the power adapter.
Sensor connections	CO2 Sensor SCD30	I2C direct connection for the SCD30 CO2 sensor. This sensor is also able to show the data through a PWM signal, however, the I2C implementation was chosen in this work.
	PM Sensor	I2C direct connection for the PM sensor HM3301.
Inputs and outputs	ESP32 indicator LED	A simple circuit to drive an indicator LED.
	Battery estimator circuit	A simple circuit to estimate the battery level using a voltage divider. Note that this is not the ideal way to estimate the battery level as it continuously drains the battery. However, it was the method chosen as it simplifies the circuit. This voltage measurement was not implemented in this work.
Miscellaneous connections	microSD	An SPI compatible connection for a microSD card module.
	RTC	An I2C compatible connection for an RTC module.
	Aux I2C	An additional I2C compatible connection for any additional 3.3 V device such as another sensor.

On the other hand, data capture is facilitated by transmitting data to a Google Sheets document with help of a custom AppScript application where every received data point is timestamped, eliminating the need for a microSD module or RTC. Furthermore, for location estimation (if needed), the project leverages the Geolocation API from Google instead of integrating a GPS module. This strategy replaces three additional electronic components (microSD, RTC, and GPS) with efficient online services.

**Fig. 2:**
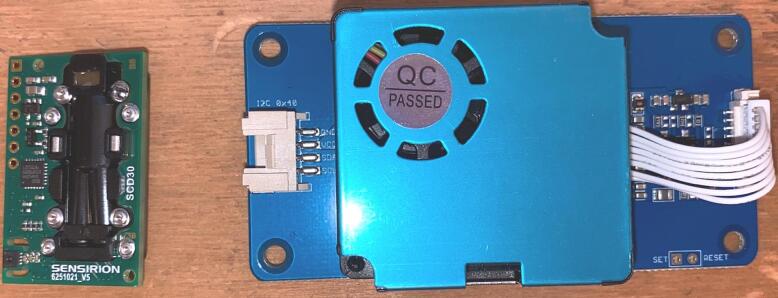
Environmental sensors, CO_2_ SCD30 (left) and Particulate Matter HM3301 (right).

AQuality32 is equipped with an SDC30 CO_2_ sensor [8] from Sensirion (refer to Fig. 2-left), which integrates a temperature and relative humidity sensor, this sensor has been tested on other works showing a remarkable performance when compared to other CO_2_ sensors [9]. Additionally, it features an HM3301 sensor [10] (refer to Fig. 2-right) from Seeed capable of detecting particulate matter across three channels, targeting different particle sizes: PM1, PM2.5, and PM10 which has also been tested on other studies showing a reliable performance [11]. The diverse resources available for ESP32 development, combined with its affordability, robust capabilities, and user-friendly interface, make this device an optimal choice for individuals seeking to customize and enhance their air quality monitoring projects. We encourage modifications to both the firmware and hardware designs, with open-source tools like KiCad and OpenSCAD which were used for circuit and case designs, fostering a collaborative and adaptable development environment.

Some possible uses for this device are:

• Environmental sciences: Mapping pollution gradients by placing sensors on several locations; Monitoring remote environments.
• Public health: Study correlations between air quality and health issues; Assessing health risks; Evaluate pollution reduction efforts.
• Indoor and outdoor air quality monitoring: Compare pollution levels on different facilities and sites; Optimize ventilation and filtration systems; Monitor personal exposure to air pollutants.

The OSF repository (https://doi.org/10.17605/OSF.IO/N9R5B) includes all the documents needed to create a copy of AQuality32, as mentioned before, everything around AQuality32 was made using free and open-source tools. Here is a description of the folders included in the repository:

*Case design source and fabrication files:* Includes the editable OpenSCAD source file with some easy-to-understand comments. This design has been optimized to keep the filling material to the minimum. It is recommended to compile the STL files from this source file since every 3D printer have their own XYZ tolerances. At the beginning of the source file there are some variables that must be adjusted depending on the specific 3D printing tolerances, the current tolerances correspond to an ANYCUBE I3 MEGA 3D PLA printer used in this project. Additionally, the folder includes the compiled STL files (upper and bottom part).

*Circuit design and fabrication files:* Includes the electronic design files made using KiCad, all the components on the design file have been referenced to make it easier to find alternative components. The design has a small label giving credit to our institution, so it is recommended to generate new production files after modifying this label. The folder also includes the production files, ready for any PCB (Printed Circuit Board) fabrication service available.

*Device firmware and web app:* Includes an example implementation project using a small Google AppScript application that takes the measurements sent by AQuality32 and then stores it on a Google Sheets document. The device’s firmware was developed with Platform IO, taking advantage of the Arduino framework and FreeRTOS and has been fully documented with doxygen comments to ease the interpretation of the code.

## 3 Design files summary

The following table contains the list of files required to reproduce AQuality32, this is the main route to the repository https://doi.org/10.17605/OSF.IO/N9R5B, the location column contains the relative location of the files: (See Table 2)

**Table 2:** Design file summary.

**Design file name**	**File type**	**Open-source license**	**Location of the file (repository folder)**
AQualityCaseBottom.stl	STL	GNU General Public License (GPL)	Case design source and fabrication files
AQualityCaseLid.stl	STL	GNU General Public License (GPL)	Case design source and fabrication files
AQualityCase.scad	CAD	GNU General Public License (GPL)	Case design source and fabrication files
AQuality.kicad_pcb	Kicad_pcb	GNU General Public License (GPL)	Circuit design and fabrication files/Design files
AQuality.kicad_sch	Kicad_sch	GNU General Public License (GPL)	Circuit design and fabrication files/Design files
AQuality32ProductionFiles.zip	ZIP	GNU General Public License (GPL)	Circuit design and fabrication files
AQualityFirware.zip	File	GNU General Public License (GPL)	Device firmware and web app
AQualityAppScript.gs	File	GNU General Public License (GPL)	Device firmware and web app/AppScript

Here is a short description of the listed files:

AQualityCaseBottom.stl: STL bottom part of the case.

AQualityCaseLid.stl: STL upper part of the case.

AQualityCase.scad: OpenSCAD design editable source code.

AQuality.kicad_pcb: KiCad editable PCB design file.

AQuality.kicad_sch: KiCad editable schematic design file.

AQuality32ProductionFiles.zip: Compressed file containing the fabrication files which can be sent to any online PCB fabrication service.

AQualityFirmware.zip: Compressed file containing the firmware.

AQualityAppScript.gs: Google AppScript code to manage the measurements of the device.

## 4 Bill of materials summary

AQuality32 was made using components easily obtained in local electronic stores. As this might be difficult depending on the country or region, the following bill of materials contains mostly part numbers for the popular online supplier DigiKey which can be found on this site using the part number on the search field. Components acquired from DigiKey might take a week or more to arrive depending on the location. Of course, alternative sites can be considered, such as Ebay or Amazon. The prices comprised on this are from February 2024 on unitary values from the suppliers. (See Table 3.)

**Table 3:** Bill of materials.

**Designator**	**Component**	**Number**	**Cost per unit (USD)**	**Total cost (USD)**	**Supplier**	**Supplier part number / Link**
POW/PROG1	Micro USB connector	1	0.46	0.46	DigiKey	609–4616-6-ND
BT1	Battery holder	1	5.34	5.34	DigiKey	36-1042P-ND
SW1	Slider switch	1	0.61	0.61	DigiKey	CKN9559-ND
PM2.5	JST 4 pin vertical connector	1	0.23	0.23	DigiKey	455-B4B-XH-A-ND
RTC1-AUXI2C1	4 pin 2.54 mm vertical socket	2	0.07	0.14	DigiKey	123-A-BL254-EG-Z04D-ND
SCD30 connector	7 pin 2.54 mm vertical socket	1	0.12	0.12	DigiKey	123-A-BL254-EG-Z07D-ND
SD1 connector	6 pin 2.54 mm vertical socket	1	0.10	0.10	DigiKey	123-A-BL254-EG-Z06D-ND
SW2-SW3	Tactile 4 pin button	2	0.18	0.36	DigiKey	CKN10880DKR-ND
U5	Battery charger	1	0.18	0.18	Octopart	TOPPOWER TP4056-42-ESOP8
U4	3.3 V LDO Voltage regulator	1	0.48	0.48	DigiKey	4518-AMS1117-3.3DKR-ND
U3	ESP32 SoM^1^	1	2.68	2.68	DigiKey	1965-ESP32-WROOM-32E-H4DKR-ND
U2	Step up IC	1	3.19	3.19	DigiKey	1188-BB-PWR-3608-ND
R6	20 k Resistor	1	0.10	0.10	DigiKey	311–20.0KFRDKR-ND
R7	22 k Resistor	1	0.10	0.10	DigiKey	311–22.0KFRDKR-ND
R8	3 k Resistor	1	0.10	0.10	DigiKey	311–3.0KERDKR-ND
R15-R16-R17	1 k Resistor	3	0.10	0.30	DigiKey	311–1.00KFRDKR-ND
R13-R14	2 k Resistor	2	0.10	0.20	DigiKey	311–2.00KFRDKR-ND
R1	33 k Resistor	1	0.10	0.10	DigiKey	311-33KERDKR-ND
R5	100 k Resistor	1	0.10	0.10	DigiKey	311-100KFRDKR-ND
R2-R3-R10-R11	10 k Resistor	4	0.10	0.40	DigiKey	311–10.0KFRDKR-ND
C2-C4-C6	4.7uF pol capacitor	3	1.14	3.42	DigiKey	P16437DKR-ND
C5	0.1uF ceramic capacitor	1	0.10	0.10	DigiKey	399-C1206C104K5RAC7800DKR-ND
C7-C8	10uF Tantalium capacitor	2	0.32	0.64	DigiKey	399–3684-6-ND
C1-C3	22uF Tantalium capacitor	2	0.47	0.94	DigiKey	478–1663-6-ND
L4R1	22uH Inductor	1	1.29	1.29	DigiKey	SRP7028A-220MDKR-ND
D1-D2	Schottky diode	2	0.19	0.37	DigiKey	3372-SS34TR-ND
Q2	P-Channel mosfet	1	0.44	0.44	DigiKey	SI2305CDS-T1-GE3DKR-ND
Q3	NPN Transistor	1	0.10	0.10	DigiKey	4878-MMBT3904DKR-ND
D4	Blue LED	1	0.23	0.23	DigiKey	732–4989-6-ND
D5	Green LED	1	0.23	0.23	DigiKey	732–4993-6-ND
D6	Red LED	1	0.23	0.23	DigiKey	732–4991-6-ND
SCD30	CO2 Sensor	1	51.95	51.95	DigiKey	1649–1098-ND
GROVE − LASER PM2.5 SENSOR (HM3301)	Particle sensor	1	32.90	32.90	DigiKey	101020613-ND
Standoff	10 mm male–female M3 plastic standoffs	4	0.53	2.12	DigiKey	36–25501-ND
Screw	M3 6 mm plastic screw	4	0.27	1.08	DigiKey	732–13704-ND
Screw	M3 6 mm metallic screw	6	0.64	3.84	DigiKey	335–1156-ND
Nut	M3 plastic nut	4	0.29	1.16	DigiKey	RPC3082-ND
18,650 Battery^2^	3.7 V 18,650 battery	1	10.00	10.00	−	−
PCB^3^	Printed Circuit Board	1	30.00	30.00	−	−
Power source^4^	5 V microUSB power adapter	1	10.00	10.00	−	−
Case^5^	PLA printed case	1	−	−	−	−
TOTAL ESTIMATED COST WITHOUT SHIPPING FEES				166.33		

1 AQuality32 uses an ESP32-WROOM-32D SoM, however, this module is now outdated and is not recommended for new developments. Hence, we recommend using the E series which is also compatible.

2 There might be some restrictions when purchasing batteries for security concerns, it must be acquired locally. A general 10 USD cost is estimated.

3 The price depends on the PCB fabrication service used, the quantity and if a stencil has been requested. So, a general 30 USD cost is estimated.

4 There are multiple options available on sites like Amazon or Ebay with different prices, a general 10 USD cost is estimated.

5 The case is optional.

## 5 Building instructions

### 5.1 Circuit assembly

**Fig. 3:**
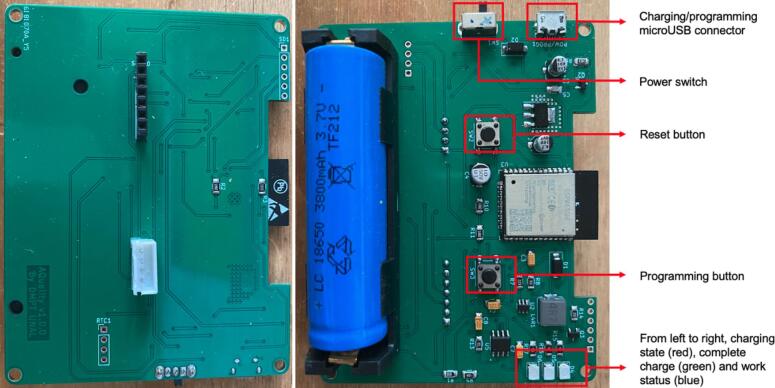
Completely assemble AQuality32 circuit TOP (left) and BOTTOM with some annotations (right).

**Fig. 4:**
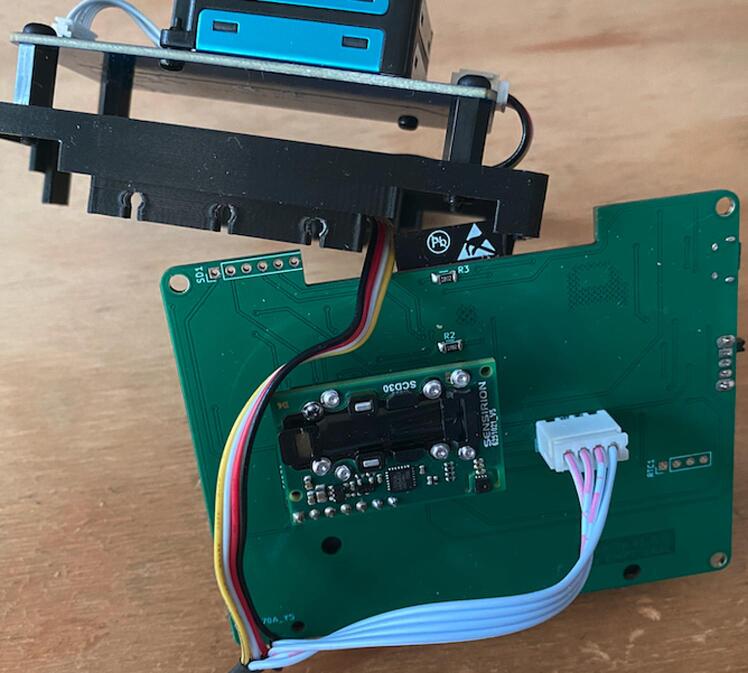
Sensor connection to PCB.

The PCB can be sent for fabrication on any online service available such as JLCPCB or PCBWAY; these services are quick and might send the production in a week. In addition to the PCB, a stencil can also be requested to ease the soldering process if a heating plate is also available, however, the components can be manually soldered. The KiCad PCB design can be used as reference to place the different components. The Fig. 3 shows a complete assembly of the circuit on both sides with some important notes on some key parts of this design:

This assembled circuit (Fig. 3) also requires the sensors, these must be connected as indicated in Fig. 4 below:

The SCD30 CO2 sensor can be connected using pin headers to the pin socket available. The HM3301 sensor must be connected to the JST connector available next to the SCD30. Note that the connector on the HM3301 is smaller than the connector on the AQuality32, hence, in this work we changed the connector on the HM3301 to make it compatible.

An essential factor to consider is the battery's impact on the device's operational duration when disconnected from a power supply. AQuality32 typically draws a current of approximately 220 mA, with 280 and 300 mA spikes when specific components are active, such as the status LED, the SCD30 and HM3301 sensors alongside Wi-Fi connectivity. Roughly assuming an average of 250 mA, a 1000 mAh 18,650 battery is projected to sustain the device during four hours under optimistic conditions. It is noteworthy that 18,650 batteries are frequently counterfeited and mislabeled, necessitating caution when procuring them.

In this point all the electronic components are connected, however, notice that there are some connectors that are not currently used, one of them allows for the connection of an SD card module and the other allows for the connection of an RTC. These two, are not in use in the current application but the user can put them into use by modifying the provided firmware. Also notice that the presented circuit is slightly different from the design provided in the repository in that the newer design has an additional I2C connector available below the RTC connector, the rest is the same. This additional connector was added later to the design to allow the connection of more sensors or peripherals.

When AQuality32 is complete like this, it is important to install a battery which will be necessary to program the device later. The battery must have some charge; however, it can be charged by installing it on the device connecting it to a 5 V, microUSB power adapter. As suggested in Fig. 3, the state of charge can be seen by checking the LEDs, red indicates that the battery is still charging and red indicates that the battery is fully charged.

### 5.2 3D printed case

The current AQuality32 case has been tailor made for the circuit to fit in. The design has been fabricated using PLA and an ANYCUBIC I3 MEGA 3D printer. The case is not mandatory for AQuality32 to work, but it is recommended for protecting the electronics. Other means of protection can also be used in case a 3D printing or even a 3D printing service is not available. However, the case presented here is not just for protection, it also allows the integration of the sensors in a single piece and slots for attaching a strap, so the device can be used for different situations. The assembly of the case is intuitive, bottom, and upper part just fit together and are secured by screw holes clearly visible to the front and back of the parts.

**Fig. 5:**
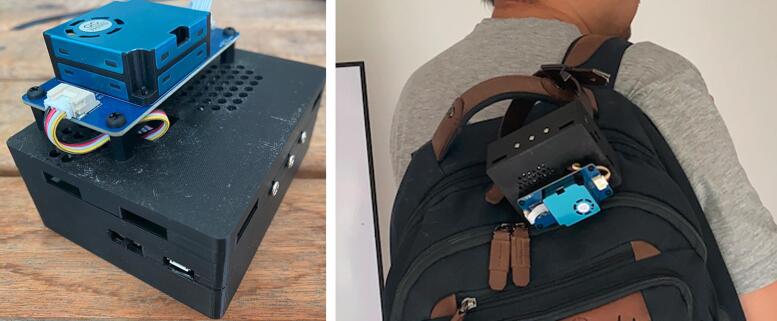
Completely assembled device (left). Alternative configuration with an additional strap (right).

Once the circuit has been assembled and the 3D case has been printed, AQuality32 can be completed, as reference, the following figure shows the completely assembled device in two configurations:

The configurations proposed on Fig. 5 allow for a simple air quality monitoring station fixed in a specific location and for a mobile air quality monitoring system. As the device requires an internet connection, the user must provide, for instance, using the phone as a hotspot in case there are not Wi-Fi connections available.

## 6 Operation instructions

### 6.1 AQuality32 programming

**Fig. 6:**
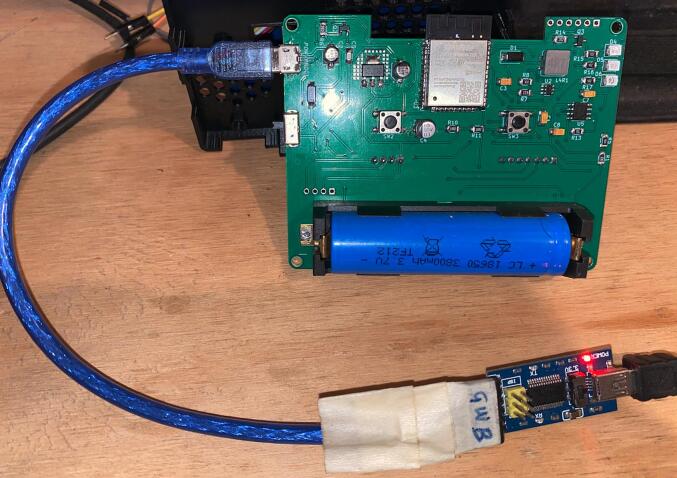
Programming connection with USB to TTL adapter.

Programming the AQuality32 device requires a USB to TTL converter, as the ESP32 module is programmed over UART. Various USB to TTL conversion options are available, many of which utilize an FTDI serial converter chip, as employed in this work. To facilitate the programming process, a standard microUSB cable was modified to connect the ground, TX, and RX lines to the FTDI module, as depicted in Fig. 6. It is imperative to utilize 3.3 V logic levels during the programming procedure:

Furthermore, to upload the firmware, AQuality32 must be placed in programming mode. This is achieved by powering off the device and powering it on while pressing the programming button (SW3 see Fig. 3). Once the device is in programming mode and connected to the PC, the firmware can be uploaded using PlatformIO in Visual Studio Code.

### 6.2 About the firmware

**Fig. 7:**
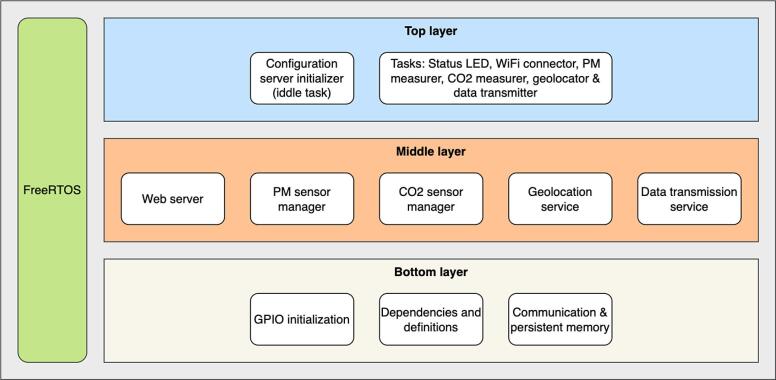
Firmware architecture used on AQuality32. FreeRTOS is depicted alongside the layers to emphasize its integration and influence across all levels of the firmware architecture.

The firmware has been fully documented using doxygen comments to ease the interpretation of the code, additionally, the firmware follows the architecture presented in Fig. 7:

The layered approach presented on Fig. 7 is a common practice in embedded system development. While adherence to this specific structure is flexible, it provides a foundation for future additions. New modules can be seamlessly integrated by placing them within the appropriate layer based on their functionality, hence, it is a good practice to follow some hierarchical order [12]. For instance, if a BMP280 pressure sensor is implemented, its dependencies should be added on the bottom layer and its reading function on the middle layer. This is not mandatory, and it is perfectly fine to put everything on the application layer directly, however, by following a layered structure the code becomes more scalable and easier to read, maintain and reuse.

### 6.3 CO2 sensor calibration

The SCD30 CO2 sensor is typically pre-calibrated by the manufacturer. However, in cases where CO2 readings fall below 400 PPM in a fresh air environment, further calibration may be necessary. This can arise due to sensor drift, environmental factors, or potential inaccuracies in the factory calibration. AQuality32′s firmware facilitates this calibration process through a dedicated macro within the “CO2_SCD30.cpp” file. While the SCD30 sensor offers an auto-calibration feature, this method can be time-consuming. Therefore, an alternative approach, as recommended by the sensor manufacturer, is implemented in AQuality32. This approach involves exposing the device to an environment with a known CO2 concentration, ideally obtained through a reliable reference measurement. If such a measurement is unavailable, a fresh air environment can be assumed to have a CO2 concentration of approximately 400 PPM. This known value is then assigned to the “refCO2” variable within the firmware. To activate this calibration routine, the DO_CALIBRATE definition within the firmware must be changed to NOT_CALIBRATE.

Upon initiating the calibration procedure, the device requires a minimum of two minutes to acquire and process data, after which the SCD30 sensor should be calibrated. To ensure normal operational following calibration, the “DO_CALIBRATE” definition must be changed again to “NOT_CALIBRATE” to prevent continuous recalibration.

### 6.4 Preparing the Google Sheets data collecting environment

Receiving AQuality32 data on a custom Google Sheets document requires a document ID which is the long string in the URL of the document, for instance:

https://docs.google.com/spreadsheets/d/<YOUR ID>/….

**Fig. 8:**
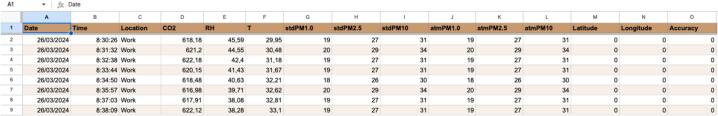
Example of logging sheet.

It is recommended to add a header to the document to easily identify the columns for analysis purposes, for instance, Fig. 8 presents an example format, along with some data captured:

The document itself cannot receive data, this is done through an AppScript application which can be created directly from the document on the extensions option. This opens an interface where the user can write code, in this case, the proposed code for capturing data from the AQuality32 is provided in the repository folder /Device firmware and web app/AppScript as ‘DataCapturer.js’. This code must be copied and pasted to the AppScript, it also contains instructions to setup the data capture application, only a few lines must be modified, for instance, at the beginning of the document the user must paste the document ID. After preparing the application it must be implemented by clicking ‘New implementation’ which then creates the application and an AppScript ID which will be used by AQuality32.

### 6.5 Preparation of geolocation services

While AQuality32 can transmit data without geolocation functionality, the integration of location information can enhance the value of the collected data. AQuality32 leverages Google Geolocation API to obtain geographical coordinates for each measurement. It is important to note that AQuality32′s firmware is specifically designed to utilize Google services; employing a different provider would necessitate modifications to the firmware. To enable geolocation, the user must possess a Google account and create a new project within the Google Cloud Console. Enabling the Geolocation API within this project is required, which involves providing billing information. However, Google provides a generous usage limit that is typically sufficient for most research applications and is reset monthly. Once enabled, the user can obtain the necessary Geolocation API key through the API and credentials section of the Google Cloud Console.

### 6.6 AQuality32 preparation

**Fig. 9:**
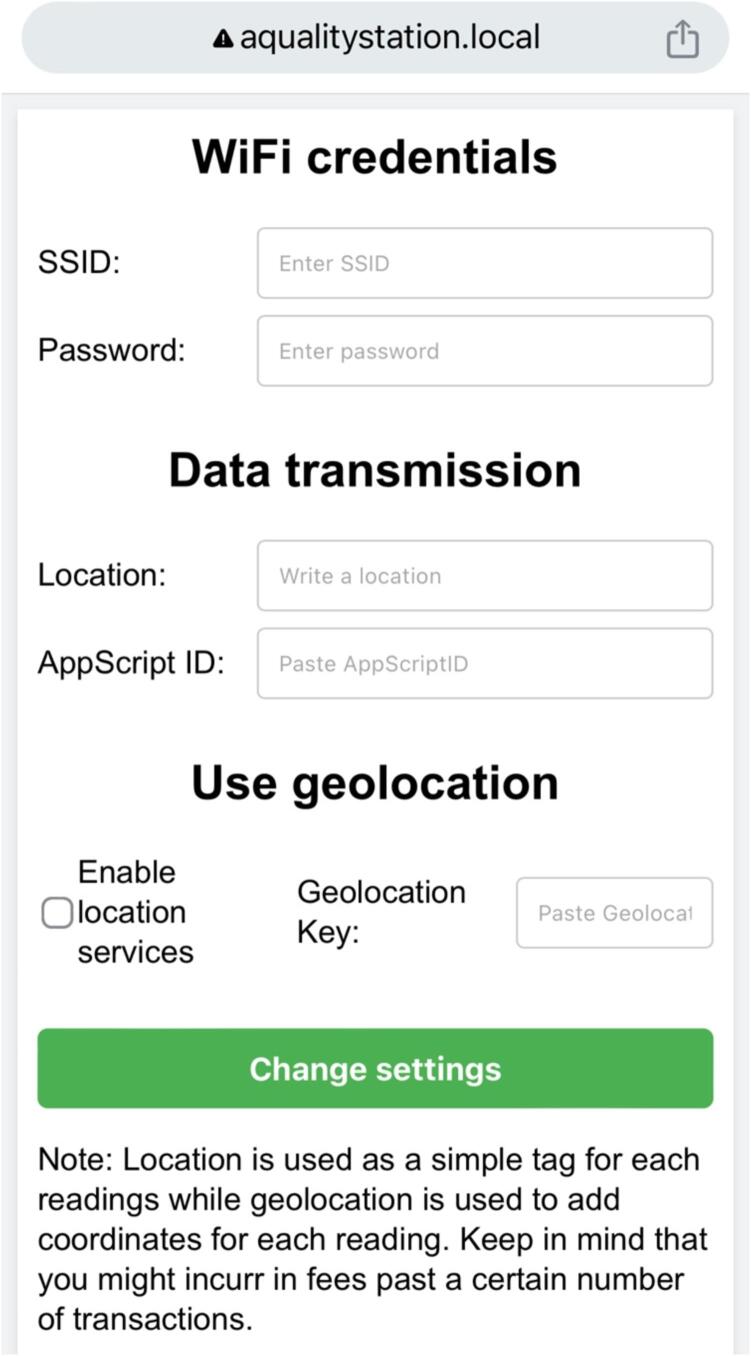
Screen capture of configuration web application running from the ESP32.

The ESP32 module in AQuality32 is capable of functioning as both an access point (AP) and a station (STA) simultaneously. This allows it to create its own Wi-Fi network (AP mode) while also connecting to an external Wi-Fi network for internet access (STA mode). If the device has been programmed successfully, it should start its own AP as ‘AQualityAP’ where the configuration web application runs. The user must access this web application either from a PC or from a phone to setup the internet access credentials and other important information. After connecting to 'AQualityAP', the user should open a web browser and navigate to 'aqualitystation.local'. In some cases, it may be necessary to use the IP address '192.168.1.200′ instead. Upon successful connection, a configuration page will be displayed, as shown in Fig. 9.

On the page presented in Fig. 9 the user must indicate the Wi-Fi credentials so that AQuality32 can access the internet, it can be a local Wi-Fi, or a hotspot created with the phone. The location field is used to easily identify where the device is sending data from or simply an informative tag for a particular experiment, the AppScript ID field allows AQuality32 to communicate with the web application and send the data to the Google Sheets document. Optionally, the geolocation option allows the device to add a coordinate to each measurement if the user also paste a valid geolocation key.

**Fig. 10:**
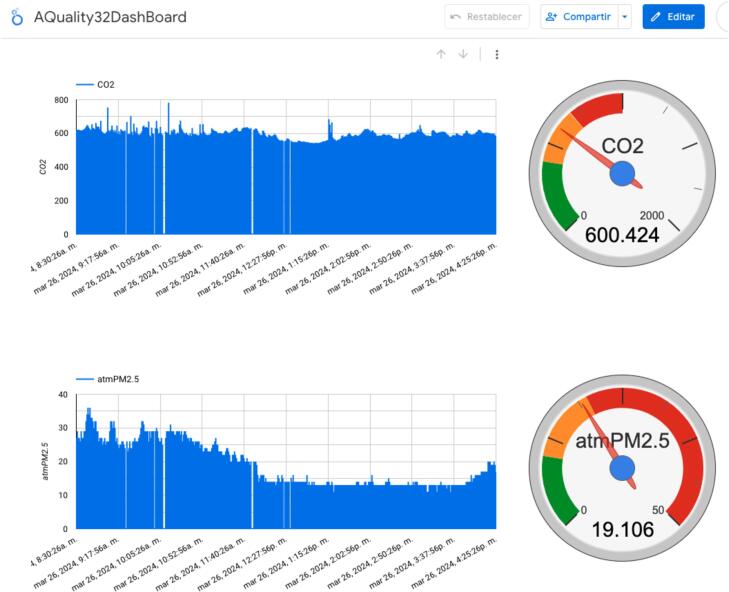
Simplistic dashboard on Looker Studio to visualize data being received.

At this point, AQuality32 should be ready to send data to the Google Sheets document. Once the data is being collected on the Google Sheets document it can be easily accessed, for instance, on a Colaboratory session to process, visualize and/or analyze data, almost in real time if necessary. Furthermore, there are other tools that can be implemented to easily visualize the data with little effort, for instance, Looker Studio, yet another useful tool from the Google platform which can be capitalized for scientific purposes at no cost. The Sheets document can be directly linked to a Looker Studio page and create a simple dashboard to present information of interest. Fig. 10 presents a simplistic example of a dashboard to show some captured data:

All elements present on Fig. 10 are natively available on the Looker Studio platform and does not require additional coding effort to make it work. This significantly facilitates the development process for anyone who wants to test the device, this approach to visualize the data is even simpler than plotting the data directly on the Sheets document.

## 7 Validation and characterization

A set of three experiments were designed to validate AQuality32 as a tool for basic environmental monitoring with extra connectivity features. These experiments were chosen to test different aspects of the device, including its communication stability, geolocation accuracy, and ability to monitor environmental conditions:

1. The **communication stability test** is crucial for ensuring the reliability of the device in capturing and transmitting data, which is essential for accurate and unbiased time-series analysis.
2. The **geolocation test** highlights the added value of incorporating location data into measurements, enabling geospatial analysis and mapping of air quality parameters.
3. The **simple environmental monitoring test** demonstrates the potential of the AQuality32 device for differentiating between contrasting environments and capturing variations in pollutant levels.

### 7.1 Communication stability test

Being able to capture data on regular intervals is crucial to properly study the time evolution of air contaminants. Not only it is important to have repeatable and quality data, but, if data is not regularly spaced in frequency, there is a risk of introducing bias when modeling. This is an issue that has been comprehensively studied in time-series before [13]. For instance, if the data obtained in the morning has a bigger size than data obtained at night due to gaps, models will be biased towards results obtained in the morning. The gap between readings must be consistent to ensure an uniform data distribution, if these gaps are abnormally large, the device is likely to have communication issues. By default, AQuality32 has been configured to send measurements in 1 + minute intervals approximately. This time interval depends on the stability of the internet network and the transaction success when the request is made, additionally, it depends on the device capturing data properly.

**Fig. 11:**
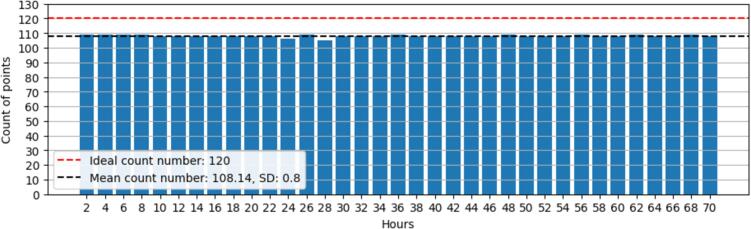
Consistency test, data points captured and sent per 2-hour intervals for 70 h.

This test assesses this communication consistency by sending data from a fixed location for several hours and counting the registered points per 2 h interval. Here, the device was left sending data for almost 3 days (70 h with location services disabled). The results are presented in Fig. 11:

As suggested by Fig. 11, the ideal data count every 2 h should be 120 points. On average it was found that the mean data count is about 108 data points every 2 h, meaning that about 6 additional data points are not captured per hour. Some of these “lost” data points might be due to communication issues which are handled with a retry approach by attempting the data transmission up to five times. Additionally, as the device takes some time to perform the data transmission, this also adds up some time which results on apparently missed data points which are not actually missed. This drawback can be minimized by modifying and tuning the firmware. Fig. 11 also shows that AQuality32 is reliable overall, it takes the same number of readings per hour, meaning that the communication is consistent if Wi-Fi communication is reliable. Any user must evaluate if this sampling rate is acceptable for their specific application, if a higher or slower sampling rate is required, the firmware must be slightly modified.

### 7.2 Geolocation test

**Fig. 12:**
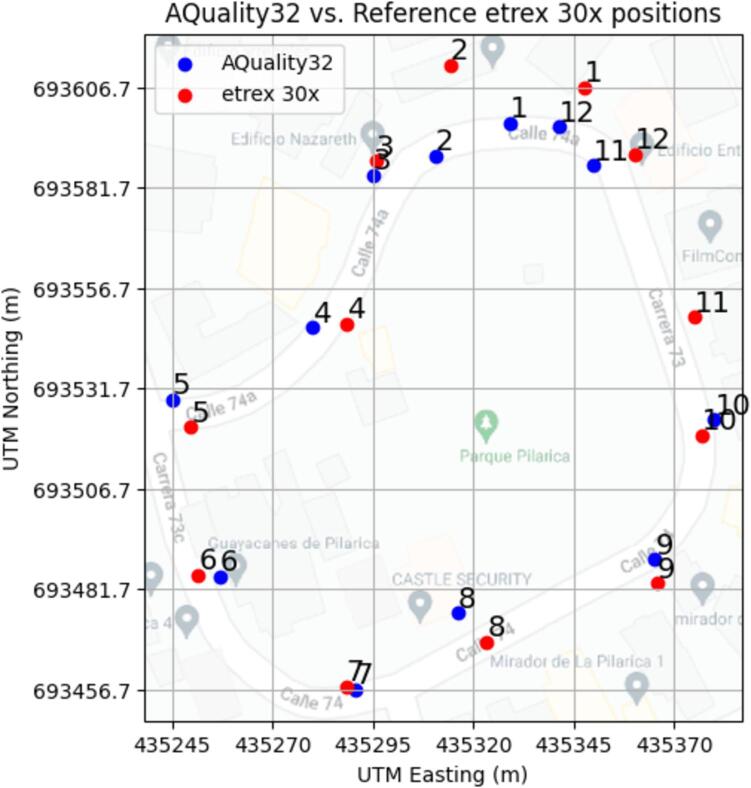
Location comparison between AQuality32 and commercially available etrex 30x from Garmin.

This test used a commercial handheld GPS navigation device from Garmin, an etrex 30x as reference to compare the coordinates obtained with those of AQuality32 which connected to a mobile hotspot provided with a smartphone. The test was performed on an urban area with high building density in Medellín (Colombia). Readings were taken on several locations with both devices until the area was surrounded starting from point 1 to 12. Then, both device’s readings were plotted on a single map tile (see Fig. 12) where each point has its associated number corresponding to the same theoretical position.

There are differences in the positions marked by AQuality32 and the etrex 30x device, and some points were relatively far from the reference. Additional metrics were obtained by measuring the Euclidean distance between pairs of points:

Mean distance between pairs of points: 13.18 m.

Standard deviation of distances between points: 12.35 m.

Median distance between pairs of points: 8.42 m.

Depending on the application, this degree of error, might be acceptable or not, for instance, on many air quality applications on cities, it is common to find air quality stations separated by hundreds or thousands of meters between them. In this scenario, having errors of about 15 m in positioning might be acceptable. Note that this exercise only serves as an example to test the positioning capabilities of AQuality32, the error found in this scenario is not fixed and depends on the surroundings and available networks. Depending on the conditions, the error might be smaller or higher.

### 7.3 Simple environment monitoring test

This simple environmental test does not intend to be a formal environmental study since this would fall outside the scope of this paper which is centered on the AQuality32 as a whole unit. However, it is important to show the operating performance of the device on a similar environmental monitoring use case. This test was designed to evaluate the capabilities of AQuality32 to differentiate between two distinct environmental settings characterized by varying levels of human activity: an urban residential area and an industrial area with high vehicular and building activity. Such differentiation was made by a qualitative analysis of the CO_2_ and PM2.5 footprints on an indoor setting.

### 7.4 Experimental setup

**Fig. 13:**
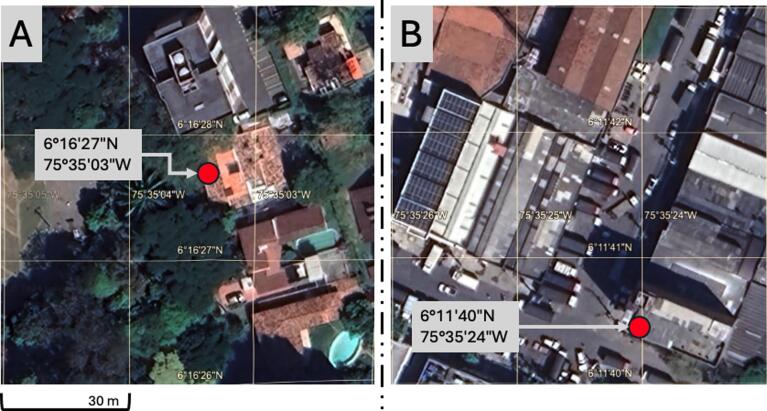
Measuring sites, urban area (A) and industrial area (B).
• **Device setting:** The experiment was carried out indoors with AQuality32 operating near an open window on both sites and connected to a 5 V micro-USB power source.
• **Data collection:** Data were collected continuously for a period of 6 h at each site, spanning from 10 am to 4 pm local time. This timeframe was selected to capture diurnal variations and potential peak periods of human activity and pollutant emissions.

• **Site selection:** Two distinct sites were chosen for this experiment: Site A, located in an urban residential area, and Site B, situated in an industrial area with high vehicular and building activity. Fig. 13 shows a satellite image of both sites.

### 7.5 Data analysis

**Fig. 14:**
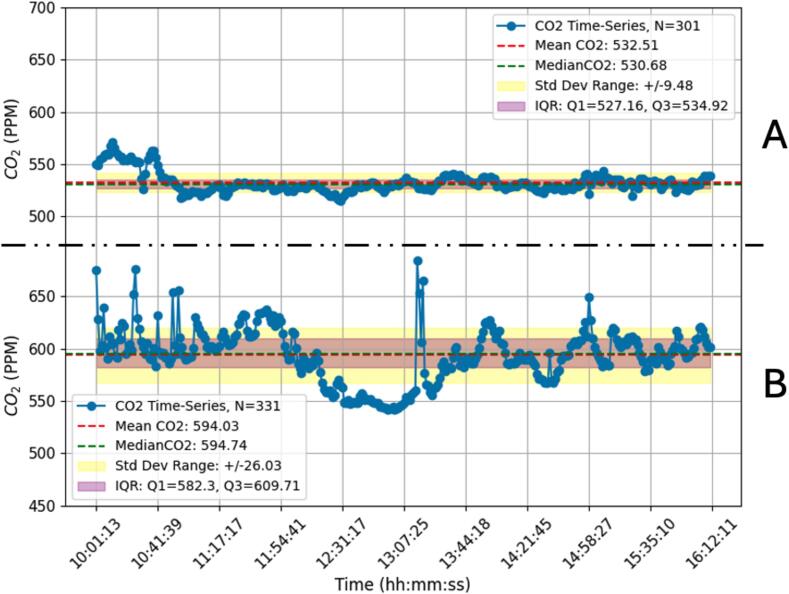
CO_2_ measurements on the urban area (A) and industrial area (B).

**Fig. 15:**
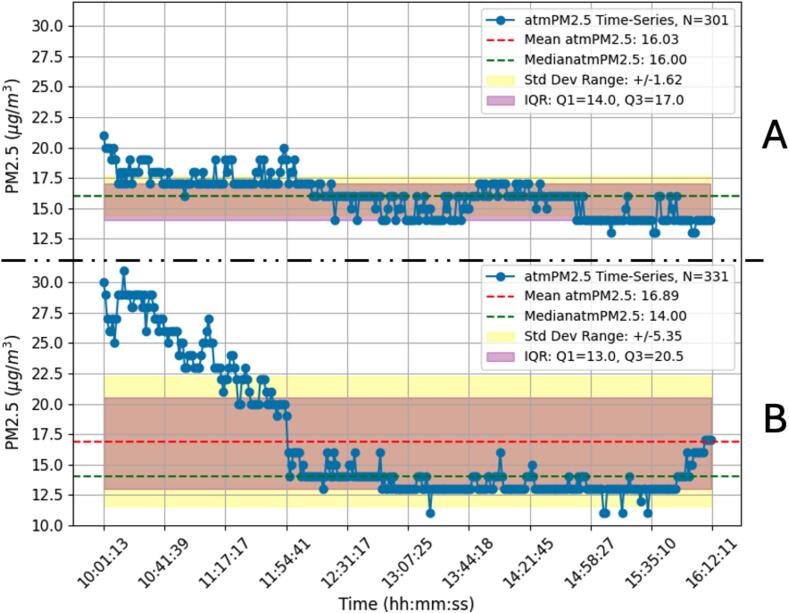
PM2.5 measurements on the urban area (A) and industrial area (B).

As this is a simple qualitative comparison between sites A and B (see Fig. 13, Fig. 14), only descriptive statistics such as mean, median, standard deviation, and interquartile range were calculated for each dataset (CO_2_ and PM2.5) to characterize the variability and central tendency of the measurements. Additionally, all this information was summarized in the form of time-series plots. Fig. 14 and Fig. 15 respectively present the CO_2_ and PM2.5 time-series collected on this test. Both figures use the same measuring scale on both sites to ease the comparison.

This simple environmental test lacks repetitions and statistical rigor, however, it clearly shows that there are apparently more pollutants present on the site with more human activity. By qualitative inspecting the plot, it is evident that the CO_2_ and PM2.5 values on the industrial area have a higher degree of variations compared to those in the urban area. Conducting longer-term monitoring campaigns spanning different seasons and weather conditions can offer a more comprehensive understanding of air quality dynamics in these environments. Furthermore, conducting parallel measurements with traditional reference instruments or other commercial air quality monitors can validate the accuracy and reliability of AQuality32′s measurements, however, as stated before, this does not intend to be a formal environmental study but a simple operating test. Continued research and collaboration are essential for advancing the capabilities and applicability of AQuality32 in addressing diverse air quality challenges and promoting public health and environmental sustainability.

### 7.6 Device limitations and future work

Moving forward, several avenues for future work and enhancements to AQuality32 can be explored. Firstly, incorporating additional sensors to measure a wider range of air quality parameters such as volatile organic compounds (VOCs), ozone (O3), nitrogen dioxide (NO2), and sulfur dioxide (SO2) could provide a more comprehensive picture of air pollution. Integration with cloud-based analytics and machine learning algorithms can enable real-time data analysis, trend identification, and predictive modeling, enhancing the device's capabilities for early warning systems and pollution forecasting. Furthermore, developing a user-friendly mobile application that takes advantage of the Bluetooth connectivity on AQuality32 would enhance its accessibility and usability. Exploring other communication protocols, such as Bluetooth Mesh, ESP-NOW, and MQTT, could further expand its connectivity options.

Local data storage might be a concern for any user in certain situations. The current focus of AQuality32 is on cloud-based data storage and visualization, but any user can modify the firmware and use the device's capability to incorporate an SD card and RTC modules which could enable local data storage, ensuring data preservation in environments with unreliable internet connectivity. This feature would increase the device's versatility and suitability for a wider range of applications, particularly in remote areas or during mobile measurements.

While AQuality32 offers significant advantages in terms of affordability, versatility, and ease of use, it also faces certain limitations that warrant consideration. One such limitation is the potential for data inaccuracies or inconsistencies due to sensor drift, calibration issues, or environmental factors affecting sensor performance. Addressing these challenges through regular sensor calibration protocols, quality control measures, and validation studies can improve data accuracy and reliability. Additionally, ensuring compatibility and interoperability with existing air quality monitoring networks and standards will be crucial for data integration, comparability, and scalability. Ongoing efforts in firmware optimization, power management, and durability enhancements will also contribute to the device's long-term performance and usability in diverse environmental conditions.

## 8 Ethics statements

This work did not involve human or animals as subjects to experiments.

## CRediT authorship contribution statement

**Daniel M. Pineda-Tobón:** Writing – original draft, Visualization, Validation, Software, Resources, Project administration, Methodology, Investigation, Funding acquisition, Formal analysis, Data curation, Conceptualization. **Albeiro Espinosa-Bedoya:** Writing – review & editing, Supervision. **Jhon W. Branch-Bedoya:** Supervision.

## Declaration of competing interest

The authors declare that they have no known competing financial interests or personal relationships that could have appeared to influence the work reported in this paper.
